# Pre-diagnostic predictors of mortality in patients with heart failure: The multi-ethnic study of atherosclerosis

**DOI:** 10.3389/fcvm.2022.1024031

**Published:** 2022-12-21

**Authors:** Mahsima Shabani, Mohammad R. Ostovaneh, Xiaoyang Ma, Bharath Ambale Venkatesh, Colin O. Wu, Harjit Chahal, Hooman Bakhshi, Robyn L. McClelland, Kiang Liu, Steven J. Shea, Gregory Burke, Wendy S. Post, Karol E. Watson, Aaron R. Folsom, David A. Bluemke, João A. C. Lima

**Affiliations:** ^1^Division of Cardiology, Department of Medicine, Johns Hopkins University, Baltimore, MD, United States; ^2^Penn State Health Milton S. Hershey Medical Center, Hershey, PA, United States; ^3^Department of Biostatistics, Bioinformatics, and Biomathematics, Georgetown University Medical Center, Washington, DC, United States; ^4^Department of Radiology, Johns Hopkins University, Baltimore, MD, United States; ^5^Office of Biostatistics Research, National Heart, Lung, and Blood Institute, National Institutes of Health, Bethesda, MD, United States; ^6^Medstar Heart and Vascular Institute, Washington, DC, United States; ^7^Inova Heart and Vascular Institute, Falls Church, VA, United States; ^8^Department of Biostatistics, University of Washington, Seattle, WA, United States; ^9^Department of Preventive Medicine, Northwestern University, Chicago, IL, United States; ^10^Department of Medicine, Vagelos College of Physicians & Surgeons, Columbia University, New York, NY, United States; ^11^Department of Epidemiology, Mailman School of Public Health, Columbia University, New York, NY, United States; ^12^Division of Public Health Sciences, Wake Forest University, Winston-Salem, NC, United States; ^13^Division of Cardiology, Department of Medicine, University of California, Los Angeles, Los Angeles, CA, United States; ^14^Division of Epidemiology and Community Health, University of Minnesota, Minneapolis, MN, United States; ^15^Department of Radiology, University of Wisconsin School of Medicine and Public Health, Madison, WI, United States

**Keywords:** prognosis, heart failure, mortality, cause of death, cumulative

## Abstract

**Background:**

There are multiple predictive factors for cardiovascular (CV) mortality measured at, or after heart failure (HF) diagnosis. However, the predictive role of long-term exposure to these predictors prior to HF diagnosis is unknown.

**Objectives:**

We aim to identify predictive factors of CV mortality in participants with HF, using cumulative exposure to risk factors before HF development.

**Methods:**

Participants of Multi-Ethnic Study of Atherosclerosis (MESA) with incident HF were included. We used stepwise Akaike Information Criterion to select CV mortality predictors among clinical, biochemical, and imaging markers collected prior to HF. Using the AUC of B-spline-corrected curves, we estimated cumulative exposure to predictive factors from baseline to the last exam before HF. The prognostic performance for CV mortality after HF was evaluated using competing risk regression with non-CV mortality as the competing risk.

**Results:**

Overall, 375 participants had new HF events (42.9% female, mean age: 74). Over an average follow-up of 4.7 years, there was no difference in the hazard of CV death for HF with reduced versus preserved ejection fraction (HR = 1.27, *p* = 0.23). The selected predictors of CV mortality in models with the least prediction error were age, cardiac arrest, myocardial infarction, and diabetes, QRS duration, HDL, cumulative exposure to total cholesterol and glucose, NT-proBNP, left ventricular mass, and statin use. The AUC of the models were 0.72 when including the latest exposure to predictive factors and 0.79 when including cumulative prior exposure to predictive factors (*p* = 0.20).

**Conclusion:**

In HF patients, besides age and diagnosed diabetes or CVD, prior lipid profile, NT-proBNP, LV mass, and QRS duration available at the diagnosis time strongly predict CV mortality. Implementing cumulative exposure to cholesterol and glucose, instead of latest measures, improves predictive accuracy for HF mortality, though not reaching statistical significance.

## Introduction

The prevalence of heart failure (HF) in the US adult population continues to rise with a reported mortality of 13.4% in 2018 (1). Thus, the early prognostication of HF has important implications at the clinical and societal level, given the high morbidity, mortality and associated resource utilization (2, 3).

Researchers have designed various risk assessment tools for assessing the prognosis of different groups of HF patients and several prognostic endpoints exist, including the Seattle Heart Failure model (4), the Metabolic Exercise test data combined with Cardiac and Kidney Indexes (5), The Heart Failure: A Controlled Trial Investigating Outcomes of Exercise TraiNing (6), and the Guiding Evidence-Based Therapy Using Biomarker Intensified Treatment (7). Moreover, several prior studies have assessed the role of cardiac structural measures and function, including myocardial strain, in the prognosis of patients with HF (8–12). Nevertheless, reported predictive factors associated with HF prognosis vary according to HF subgroup (HF with reduced ejection fraction [HFrEF] and HF with preserved ejection fraction [HFpEF]) and whether prognostic factors were measured before or after incident HF. To date, few studies have assessed whether variables measured exclusively before the diagnosis of HF would predict subsequent cardiovascular (CV) mortality and whether the time-weighted average of these variables would improve the predictive performance.

The Multi-Ethnic Study of Atherosclerosis (MESA) provides the infrastructure to evaluate determinants of HF prognosis based on factors assessed years before the development of clinically manifested disease. The availability of many CV imaging biomarkers in this cohort, and in particular cardiac MRI (CMR), makes MESA a unique cohort for studying predictors of HF prognosis. These imaging biomarkers, along with other demographic and laboratory markers, are independently associated with incident HF and other CV events (13–18). In this study, we assessed the value of clinical, biochemical, and imaging markers obtained prior to the diagnosis of HF, including the time-weighted average of their past exposures, to predict the prognosis of participants with incident clinically evident HF in a multi-ethnic population. Performance of the prediction models were compared between participants with HFrEF and HFpEF.

## Materials and methods

### Study population

The baseline MESA cohort consisted of 6,814 participants aged 45–84 years across 6 field centers in the US from four racial/ethnic groups (White, Chinese, Black and Hispanic) free of known clinical cardiovascular disease (CVD) at baseline (19). The detailed design of MESA is described elsewhere (19). Institutional review boards at each field centers approved the study protocol, and all participants gave written informed consent. Participants who developed HF (landmark of study) at any time after baseline exam (2000–2002) were included in this analysis. Participants were categorized as HFpEF or HFrEF based on ejection fraction (EF) greater or lower than 45% based on clinical studies obtained at the time of initial HF diagnosis. Participants without available EF measured at the time of HF (*n* = 111) were excluded from the HF subtype-specific analysis.

### Predictors of mortality

We selected demographic, clinical, biochemical, and imaging data from the most recent MESA exam prior to the HF diagnosis (exams one to five). The age and EF (to categorize HFpEF and HFrEF) variables were collected at the time of HF diagnosis (landmark). Missing values for pre-HF diagnosis risk factors with less than 10% missingness were imputed using logistic regression or mean estimation methods on a variable-based approach. We included cumulative exposure to modifiable risk factors (prior to HF diagnosis) including waist circumference, body mass index (BMI), serum fasting glucose, low-density (LDL) and high-density (HDL) lipoprotein cholesterol, total cholesterol, triglycerides, systolic (SBP) and diastolic (DBP) blood pressure, and glomerular filtration rate (GFR). Participants with only one measurement of these risk factors prior to HF were excluded from the cumulative analysis. We estimated the subject-specific trajectories for variables that had repeated measures through each exam, using cubic spline-based mixed-effect models (Supplementary Figure 1) (20). We used this method due to its ability to develop a non-linear exposure curve over the exposure period. The cumulative exposure was calculated as the area under curve from baseline until HF diagnosis divided by the time interval in years. The Best Linear Unbiased Prediction (BLUP) is used to predict individual trajectories when the datapoints are at irregular intervals (21). The AUC method has been used in previous studies to estimate the cumulative exposure to risk factors (20, 22). Other non-cumulative variables utilized in the analysis included: demographics (age, sex, race/ethnicity), smoking (never, former, ever), history of myocardial infarction (MI), atrial fibrillation (Afib), resuscitated cardiac arrest (RCA), chronic obstructive pulmonary disease (COPD), transient ischemic attack (TIA), stroke, ankle-brachial index (ABI), medication use (statin, aspirin, nitrate, beta-blocker, ACE inhibitor, angiotensin receptor blocker, anti-coagulants, and diuretics), electrocardiogram variables (PR, QRS, and QTc duration), cardiac magnetic resonance imaging-derived left atrial (LA) and ventricular (LV) structure and function (LV end-diastolic mass index [LVM], LV end-diastolic and end-systolic volume index, LV EF, LA active, passive, and total EF), and coronary artery calcification Agatston score (CAC). History of MI, Afib, RCA, COPD, TIA, and stroke was defined as occurrence of the respective event any time between the baseline exam and time of HF diagnosis. The EF at the time of HF diagnosis was available for 264 participants (70.4% of all participants with a HF event).

### Endpoint assessment

Since baseline, MESA staff have telephoned participants to inquire about hospital admissions, selected outpatient diagnoses and procedures every 9–12 months. Medical records were sought for all eligible CV events and reviewed by two independent physicians for validation. HF diagnosis was defined as either a definite or a probable diagnosis in symptomatic participants. Probable HF classification required the record to contain evidence of HF symptoms and a physician-made diagnosis and use of HF-specific medication. Definite HF required evidence of either (a) chest X-ray derived pulmonary congestion/edema or (b) imaging-based LV dilatation or dysfunction, or diastolic dysfunction. Asymptomatic participants were not considered as meeting an endpoint in this cohort.

Participants were followed for mortality after incident HF. MESA physician reviewers classified cardiovascular deaths based on the death certificate, recent hospital records, and sometimes informant interviews and surveys of participants’ physicians. CV deaths, the outcome of this report, were categorized as definite or possible based on recorded underlying cause of death, history, and symptoms at the time of death.

### Statistical analysis

**FIGURE 1:**
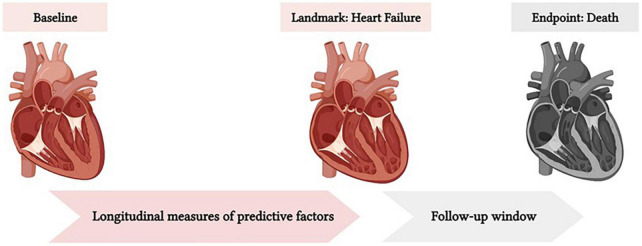
The landmark of the survival analysis for this study was set at the time of HF diagnosis. The baseline for cumulative analysis was MESA exam 1 (Time Zero). The prediction window was from landmark to death or maximum follow-up time. The cumulative exposure to predictive factors from baseline to the landmark was used to predict mortality after heart failure.

The data are presented as means (standard deviation) or numbers (%). We used landmark analysis to predict mortality in the follow-up window after the diagnosis of HF as our landmark (Figure 1), using latest measures of variables or their cumulative exposure in the observation window (from baseline MESA exam to the latest exam before the landmark) (23). We used the Akaike Information Criterion (AIC)-based goodness of fit method to select the best predictors, among 45 variables of CV mortality after incident HF. Age-adjusted associations were assessed using multivariable Fine and Gray competing risk survival regression analysis. In time-to-event analyses, the time origin (T_0_) was set as the time of HF incidence and non-CV mortality was considered as a competing risk. We constructed two Fine and Gray competing risk regression models with modifiable risk factors included as either cross-sectional (latest MESA exam before incident HF) or cumulative prior exposure (from baseline to the latest MESA exam before incident HF) values. After selection of the models with maximum likelihood, the models were applied on both groups of HFrEF and HFpEF. The sub-distribution HR (sHR) was reported for both CV mortality and non-CV mortality to facilitate the interpretation of results. Prediction performance of regression models were compared by area under the ROC curve (AUC) analysis and Harrell’s C-index, and predictive accuracy was evaluated by the Brier score (24). Brier score provides an estimation of the squared difference between actual outcome and model prediction, with values ranging from 0 (perfect model) to a maximum value less than 1 depending on the outcome incidence. Data analysis was performed using R programing platform (v 3.6.3).

## Results

A total of 375 participants who developed HF after the baseline MESA exam (132 with HFrEF, 132 with HFpEF, 111 with unidentified EF) were included (Supplementary Figure 2), 340 (90.6%) of whom were hospitalized for HF at the time of diagnosis. For the cumulative analysis we included 317 participants who had more than one measurement of modifiable risk factors prior to incident HF.

**FIGURE 2:**
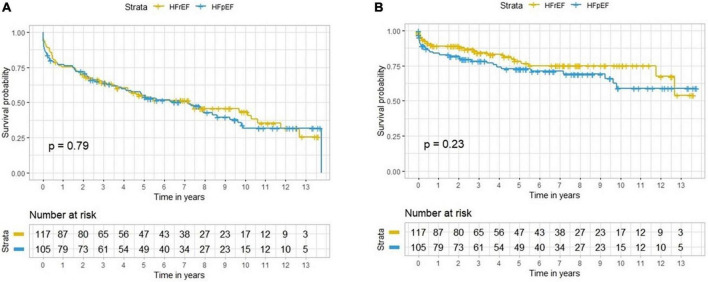
Survival curves for all-cause mortality **(A)** and CV death **(B)** in participants with HFrEF and HFpEF.

A descriptive population distribution in participants with incident HFrEF vs. HFpEF is available in Table 1. During 1,785 person-years of follow-up (minimum of 0.6 year to 14.4 years), 212 participants with incident HF died, 98 (46.2%) due to CV causes and 114 (53.8%) of non-CVD causes. The overall mortality rate per follow-up time was similar between HFrEF (60.6%) and HFpEF (57.6%) groups (*p* = 0.71). Among the HFrEF participants, 40 (30.3%) died of CVD and 40 (30.3%) died of non-CV causes, whereas in HFpEF participants, 30 (22.7%) died of CVD and 46 (34.8%) died of non-CV causes (*p* = 0.37). Similar to overall mortality, there was no significant difference in the hazard of CV mortality in the HFrEF compared to the HFpEF group (HR = 1.27, *p* = 0.23) (Figure 2).

**TABLE 1:** Distribution of variables prior to HF diagnosis in participants with CV vs. non-CV death and total population, stratified by HF subcategories (HF with reduced ejection fraction [HFrEF] vs. HF with preserved ejection fraction [HFpEF]).

	HFrEF				HFpEF			
		
	CV death	Non-CV death	Total	*P*-value	CV death	Non-CV death	Total	*P*-value
								
	40	40	132		30	46	132	
Age (years)	75.5 (8.6)	74.4 (7.9)	72.1 (9.6)	**<0.001**	76.0 (9.3)	77.9 (7.4)	74.4 (9.1)	**<0.001**
Male gender	31 (77.5%)	29 (72.5%)	94 (71.2%)	0.438	16 (53.3%)	24 (52.2%)	64 (50.0%)	0.540
**Race**								
White	13 (32.5%)	16 (40.0%)	55 (41.7%)	0.315	12 (40.0%)	26 (56.5%)	57 (43.2%)	0.226
Chinese American	0 (0.0%)	1 (2.5%)	2 (1.5%)		3 (10.0%)	3 (6.5%)	15 (11.4%)	
African American	21 (52.5%)	13 (32.5%)	52 (39.4%)		6 (20.0%)	11 (23.9%)	31 (23.5%)	
Hispanic	6 (15.0%)	10 (25.0%)	23 (17.4%)		9 (30.0%)	6 (13.0%)	29 (22.0%)	
BMI (Kg/m^2^)	28.6 (5.3)	28.2 (5.1)	28.8 (4.9)	0.457	30.4 (6.7)	29.5 (6.8)	30.3 (6.5)	0.437
Waist circumference (cm)	100.3 (12.8)	101.1 (12.6)	101.8 (13.0)	0.734	107.9 (18.4)	105.6 (16.3)	105.9 (16.0)	0.813
**Smoking**								
Former smoker	18 (45.0%)	28 (70.0%)	74 (56.1%)	0.245	15 (50.0%)	27 (58.7%)	64 (48.5%)	0.227
Current smoker	7 (17.5%)	4 (10.0%)	20 (15.2%)		3 (10.0%)	5 (10.9%)	12 (9.1%)	
Education higher than 12th grade	30 (75.0%)	31 (79.5%)	106 (80.9%)	0.364	22 (73.3%)	32 (69.6%)	96 (72.7%)	0.826
History of CVD	16 (40.0%)	18 (45.0%)	51 (38.6%)	0.475	15 (50.0%)	23 (50.0%)	62 (47.0%)	0.719
History of Afib	6 (15.0%)	11 (27.5%)	23 (17.6%)	0.129	3 (10.7%)	10 (23.3%)	20 (15.9%)	0.257
History of MI	16 (40.0%)	15 (37.5%)	43 (32.6%)	0.167	12 (40.0%)	15 (32.6%)	43 (32.6%)	0.559
History of RCA	7 (17.5%)	4 (10.0%)	12 (9.1%)	**0.035**	9 (30.0%)	4 (8.7%)	13 (9.8%)	**<0.001**
History of COPD	10 (25.0%)	9 (22.5%)	23 (17.4%)	0.057	7 (23.3%)	10 (21.7%)	20 (15.2%)	**0.026**
History of TIA	7 (17.5%)	4 (10.0%)	11 (8.3%)	**0.010**	5 (16.7%)	4 (8.7%)	11 (8.3%)	0.111
History of stroke	8 (20.0%)	6 (15.0%)	15 (11.4%)	**0.018**	7 (23.3%)	6 (13.0%)	17 (12.9%)	0.102
SBP (mmHg)	137.1 (25.4)	130.9 (19.9)	133.2 (21.1)	0.464	137.4 (22.6)	136.6 (22.4)	136.5 (22.7)	0.962
DBP (mmHg)	72.0 (13.1)	68.8 (9.5)	71.3 (11.5)	0.641	68.2 (13.0)	69.7 (10.7)	69.4 (10.9)	0.605
FBG (mg/dL)	106.0 (26.6)	112.7 (44.4)	105.0 (32.0)	0.087	113.8 (37.0)	103.0 (24.9)	108.5 (33.0)	0.556
Triglyceride (mg/dL)	124.1 (74.6)	106.5 (45.8)	116.3 (65.9)	0.911	132.7 (99.0)	102.2 (46.7)	125.2 (84.9)	0.106
LDL (mg/dL)	111.0 (33.7)	99.6 (30.7)	106.4 (34.0)	0.270	90.9 (32.3)	95.1 (31.8)	98.2 (32.7)	0.233
HDL (mg/dL)	47.3 (11.5)	47.9 (11.2)	50.0 (13.3)	0.108	50.0 (13.8)	52.3 (16.1)	51.7 (16.3)	0.882
Cholesterol (mg/dL)	183.0 (38.6)	168.8 (34.4)	179.6 (40.6)	0.105	167.5 (37.5)	167.8 (36.2)	174.3 (37.9)	0.114
**DM status**								
IFG	7 (17.5%)	7 (17.5%)	19 (14.4%)	0.134	10 (33.3%)	12 (26.1%)	30 (22.7%)	0.056
Untreated DM	0 (0.0%)	4 (10.0%)	6 (4.5%)		2 (6.7%)	2 (4.3%)	4 (3.0%)	
Treated DM	13 (32.5%)	11 (27.5%)	34 (25.8%)		11 (36.7%)	10 (21.7%)	41 (31.1%)	
GFR (mL/min)	74.69 (23.2)	69.45 (24.8)	73.04 (21.2)	0.505	64.4 (20.5)	75.3 (26.6)	71.8 (23.3)	0.093
CRP (mg/L)	5.8 (3.5)	9.3 (25.1)	6.5 (13.9)	0.573	6.6 (6.3)	6.2 (5.4)	6.4 (5.0)	0.480
Fibrinogen (mg/dL)	413.5 (65.2)	416.1 (134.7)	406.6 (88.2)	0.560	446.7 (66.9)	416.5 (76.6)	421.6 (63.5)	0.075
Troponin (log)	–4.6 (0.3)	–4.6 (0.2)	–4.6 (0.3)	0.946	–4.46 (0.5)	–4.54 (0.4)	–4.58 (0.36)	**0.008**
NT-proBNP (pg/mL)	574.4 (810.8)	425.9 (632.2)	434.0 (605.6)	0.092	466.0 (718.0)	299.6 (249.7)	302.0 (400.4)	**0.041**
CAC (Agatson score)	761.3 (1033.9)	940.2 (1144.6)	723.6 (937.8)	0.316	690.2 (797.3)	799.0 (1196.7)	712.8 (944.7)	0.659
ABI	1.02 (0.2)	1.04 (0.2)	1.08 (0.2)	**0.011**	1.05 (0.2)	1.08 (0.1)	1.09 (0.1)	0.313
Anti-hypertensive use	27.0 (67.5%)	27.0 (67.5%)	87.0 (65.9%)	0.892	25 (83.3%)	38 (82.6%)	103 (78.0%)	0.290
Statin use	10 (25.0%)	18 (45.0%)	41 (31.1%)	0.074	10 (33.3%)	19 (41.3%)	58 (43.9%)	0.235
Aspirin use	17 (42.5%)	17 (42.5%)	56 (42.4%)	0.999	17 (56.7%)	20 (43.5%)	62 (47.0%)	0.477
ACEI use	12 (30.0%)	10 (25.0%)	33 (25.0%)	0.624	10 (33.3%)	12 (26.1%)	43 (32.6%)	0.471
ARB use	5 (12.5%)	4 (10.0%)	19 (14.4%)	0.421	4 (13.3%)	6 (13.0%)	21 (15.9%)	0.602
ACEI + Diuretic use	1 (2.5%)	9 (22.5%)	14 (10.6%)	**0.010**	4 (13.3%)	7 (15.2%)	15 (11.4%)	0.410
ARB + Diuretic use	2 (5.0%)	7 (17.5%)	16 (12.1%)	0.215	4 (13.3%)	8 (17.4%)	19 (14.4%)	0.769
Nitrate use	1 (2.5%)	6 (15.0%)	13 (9.8%)	0.150	2 (6.7%)	8 (17.4%)	16 (12.1%)	0.343
Diuretic use	13 (32.5%)	16 (40.0%)	46 (34.8%)	0.715	10 (33.3%)	22 (47.8%)	48 (36.4%)	0.122
Beta-blocker use	6 (15.0%)	11 (27.5%)	28 (21.2%)	0.393	9 (30.0%)	13 (28.3%)	37 (28.0%)	0.950
Anti-coagulant use	1 (2.5%)	8 (20.0%)	20 (15.2%)	**0.028**	3 (10.0%)	9 (19.6%)	20 (15.2%)	0.509
PR duration (ms)	182.6 (44.3)	175.2 (14.9)	175.8 (29.2)	0.492	175.7 (14.7)	172.6 (26.1)	171.6 (23.9)	0.055
QRS duration (ms)	106.5 (18.5)	104.2 (14.3)	104.8 (16.5)	0.746	100.9 (4.6)	100.9 (14.7)	97.7 (11.5)	**<0.001**
QTc duration (ms)	437.4 (19.0)	436.7 (20.9)	438.4 (24.2)	0.851	431.9 (15.0)	435.8 (15.7)	431.9 (18.3)	0.172
LVM (g/m^2^)	99.3 (21.9)	95.6 (21.9)	94.1 (20.7)	**0.035**	91.3 (13.5)	82.9 (12.4)	85.3 (13.1)	0.051
LV EDV (mL/m^2^)	81.3 (16.7)	76.4 (16.8)	78.7 (18.1)	0.309	70.5 (7.6)	69.9 (12.8)	70.2 (11.9)	0.811
LV ESV (mL/m^2^)	35.6 (15.0)	30.5 (11.8)	33.0 (14.0)	0.066	25.6 (7.3)	25.8 (8.8)	25.5 (8.3)	0.751
LV EF (%)	58.6 (10.0)	61.7 (7.7)	60.0 (9.1)	0.272	65.1 (8.0)	65.0 (7.8)	65.2 (7.2)	0.751
LA min volume (mL/m^2^)	25.1 (7.3)	22.4 (6.9)	23.8 (7.8)	0.279	23.1 (6.6)	23.6 (5.9)	23.0 (6.2)	0.537
LA max volume (mL/m^2^)	41.3 (8.9)	37.1 (10.3)	39.4 (10.2)	0.197	37.7 (7.4)	39.1 (8.5)	39.0 (8.4)	0.901
LA active EF (%)	29.5 (6.0)	28.6 (6.8)	29.2 (6.7)	0.720	28.7 (7.9)	28.8 (6.2)	30.1 (7.1)	0.146
LA passive EF (%)	16.2 (3.9)	16.9 (4.9)	16.9 (4.3)	0.843	16.9 (4.3)	16.8 (4.3)	17.5 (5.2)	0.648
LA total EF (%)	40.6 (6.8)	40.2 (5.7)	40.6 (7.2)	0.963	39.5 (9.0)	40.7 (6.0)	42.0 (7.5)	**0.029**

BMI, body mass index; CVD, cardiovascular diseases; Afib, atrial fibrillation; MI, myocardial infarction; RCA, resuscitated cardiac arrest; COPD, chronic obstructive pulmonary disease; TIA, transient ischemic attack; SBP, systolic blood pressure; DBP, diastolic blood pressure; FBG, fasting blood glucose; LDL, low-density lipoprotein; HDL, high-density lipoprotein; DM, diabetes mellitus; IFG, impaired fasting glucose; GFR, glomerular filtration rate, CAC, coronary artery calcium, ABI, ankle-brachial index; ACEI, angiotensin-converting-enzyme inhibitors; ARB, angiotensin receptor blockers; LV, left ventricle; LA, left atrium; EDM, end-diastolic mass; EDV, end-diastolic volume; ESV, end-systolic volume; EF, ejection fraction. *P* values are provided for the comparison of variables between participants with CV death, non-CV death, or censored group (not shown here due to redundancy). Bold values represent *p* value < 0.05.

Participants with HFrEF who died from CV causes were older, were more likely to have history of resuscitated cardiac arrest (RCA), TIA, and stroke, lower ABI, and higher LVM, and a lower cumulative exposure to HDL cholesterol levels compared to HFrEF participants who were alive at the end of follow-up (Table 1). Participants with HFpEF who died from CV causes were older, were more likely to have RCA, and COPD prior to HF, had longer QRS duration, higher troponin and NT-proBNP levels, and lower total LA EF, compared to those who were alive at the end of follow-up (Table 1 and Supplementary Table 1).

Table 2 lists the sHR for prediction of CV mortality for variables selected based on backward stepwise regression using AIC criteria. Predictors of CV mortality in non-cumulative analysis included age (sHR:1.06 per year, 95% CI: [1.03–1.09]), history of RCA (5.2 [3.1–8.8]), diabetes (1.7 [1.1–2.7]), QRS duration in ECG (1.3 per SD [1.1–1.6]), total cholesterol (1.4 per SD [1.1–1.7]), HDL cholesterol (0.8 per SD [0.6–0.9]), LVM (1.2 per SD [1.1–1.5]), NT-proBNP (1.3 per SD [1.1–1.5]), and statin use (0.6 [0.4–1.0]).

**TABLE 2:** The association between time-to-CV mortality and selected variables in participants with incident HF.

Model 1						

Predictor	All HF (375 subjects)		HFrEF (*n* = 132)		HFpEF (*n* = 132)	
			
	sHR (95% CI)					
			
	CV death	Other death	CV death	Other death	CV death	Other death
Age (years)	**1.06 (1.03–1.09)**	**1.03 (1.01–1.05)**	**1.07 (1.03–1.11)**	1.03 (0.99–1.06)	1.03 (0.98–1.09)	**1.06 (1.02–1.10)**
History of RCA	**5.25 (3.14–8.79)**	1.01 (0.51–1.99)	**3.62 (1.52–8.64)**	1.61 (0.41–6.28)	**9.89 (4.01–24.39)**	0.83 (0.26–2.64)
DM	**1.75 (1.11–2.75)**	0.94 (0.62–1.43)	1.72 (0.78–3.80)	1.04 (0.49–2.18)	1.33 (0.58–3.02)	0.88 (0.39–1.97)
QRS duration (per SD)	**1.28 (1.05–1.56)**	1.08 (0.92–1.27)	1.27 (0.94–1.73)	0.88 (0.62–1.24)	1.07 (0.67–1.71)	**1.38 (1.02–1.87)**
HDL (per SD)	**0.77 (0.61–0.97)**	1.04 (0.85–1.27)	0.66 (0.42–1.03)	0.93 (0.61–1.40)	0.88 (0.61–1.27)	0.98 (0.70–1.36)
Total cholesterol (per SD)	**1.38 (1.10–1.73)**	**0.76 (0.61–0.96)**	**1.42 (1.01–2.01)**	0.74 (0.52–1.06)	1.03 (0.60–1.76)	0.75 (0.51–1.09)
LVM (per SD)	**1.21 (1.00–1.47)**	0.88 (0.72–1.09)	**1.25 (1.00–1.59)**	0.98 (0.68–1.42)	**2.27 (1.37–3.76)**	**0.70 (0.49–0.99)**
NT-proBNP (per SD)	**1.31 (1.14–1.49)**	0.86 (0.63–1.15)	1.12 (0.88–1.42)	0.90 (0.51–1.60)	**1.61 (1.35–1.92)**	0.83 (0.53–1.28)
Statin use	0.65 (0.41–1.03)	1.07 (0.70–1.63)	0.78 (0.34–1.76)	1.60 (0.78–3.31)	0.50 (0.21–1.15)	0.58 (0.29–1.15)

**Model 2**						

	**All HF (318 subjects)**		**HFrEF (*n* = 105)**		**HFpEF (*n* = 117)**	
			
	**sHR (95% CI)**					

Age (years)	**1.05 (1.02–1.09)**	**1.03 (1.01–1.06)**	**1.09 (1.03–1.15)**	1.02 (0.98–1.07)	1.03 (0.97–1.09)	**1.09 (1.03–1.15)**
History of RCA	**4.76 (2.67–8.50)**	1.19 (0.58–2.46)	**3.15 (1.18–8.39)**	1.97 (0.51–7.62)	**17.48 (4.95–61.69)**	0.99 (0.30–3.27)
History of MI	1.50 (0.89–2.54)	1.01 (0.99–1.02)	1.40 (0.53–3.66)	1.35 (0.64–2.85)	0.82 (0.28–2.43)	0.85 (0.37–1.94)
QRS duration (per SD)	**1.28 (1.01–1.62)**	1.11 (0.92–1.33)	1.17 (0.82–1.68)	0.89 (0.60–1.32)	1.30 (0.82–2.05)	**1.39 (1.02–1.87)**
Cumulative exposure to total cholesterol (per SD)	**1.35 (1.06–1.73)**	**0.72 (0.58–0.89)**	**1.51 (1.00–2.31)**	**0.63 (0.43–0.92)**	1.18 (0.68–2.04)	**0.69 (0.50–0.96)**
Cumulative exposure to glucose (per SD)	**1.27 (1.02–1.59)**	1.00 (0.81–1.25)	0.86 (0.47–1.58)	1.14 (0.76–1.70)	1.39 (0.99–1.95)	0.92 (0.15–5.66)
LVM (per SD)	**1.49 (1.15–1.94)**	**0.75 (0.61–0.93)**	**1.49 (1.14–1.96)**	0.77 (0.51–1.14)	**2.18 (1.19–3.99)**	0.73 (0.50–1.05)
NT-proBNP (per SD)	1.13 (0.91–1.42)	0.84 (0.50–1.40)	1.04 (0.82–1.32)	0.96 (0.41–2.25)	1.58 (1.29–1.95)	0.91 (0.64–1.30)
Statin use	0.66 (0.40–1.10)	1.12 (0.72–1.73)	0.92 (0.33–2.52)	1.78 (0.81–3.91)	0.64 (0.27–1.49)	0.52 (0.25–1.11)

Fine and Gray competing risk models and backward stepwise covariate selection by AIC criteria were used with latest measures only (Model 1) and time-weighted average of variables (Model 2) (Cardiovascular death: failure event of interest, other death: competing risk). SD, standard deviation; RCA, resuscitated cardiac arrest; SBP, systolic blood pressure; LDL, low-density lipoprotein; HDL, high-density lipoprotein; DM, diabetes mellitus; ABI, ankle-brachial index; ARB, angiotensin receptor blockers; LV, left ventricle; EDM, end-diastolic mass. Bold values represent *p* value < 0.05.

This model had the lowest prognostic error for CV mortality (Log Likelihood: –502.4) with an AUC of 0.74 and a Brier score of 0.29 for prediction of CV death in HF patients (Table 3A). When applied to the groups with HFrEF and HFpEF separately, similar prediction performances and accuracies were noted (*p* = 0.35 for difference in AUC, *p* = 0.32 for difference in Brier score, Tables 3B, C). Univariable and age-adjusted competing risk models for CV death with each covariate used for variable selection are available in Supplementary Table 2.

**TABLE 3:** Model comparison with model-specific area-under-curve (AUC) and Brier score and delta score between pairs of models in all HF subjects (A), model 1 in HFrEF vs. HFpEF (B), and model 2 in HFrEF vs. HFpEF (C).

Models	AUC (95% CI)	Brier (95% CI)	C-index
**(A)**			
1	0.741 (0.575–0.907)	0.291 (0.222–0.360)	74.5
2	0.820 (0.671–0.968)	0.289 (0.221–0.358)	78.4
Difference	0.078 (0.013–0.143) *p* = 0.02	–0.002 (–0.004 to 0.001) *p* = 0.26	

**Model 1**	**AUC (95% CI)**	**Brier (95% CI)**	**C-index**

**(B)**			
HFrEF	0.751 (0.675–0.828)	0.286 (0.218–0.353)	70.9
HFpEF	0.791 (0.716–0.867)	0.290 (0.220–0.360)	78.9
Difference	0.04 (–0.044 to 0.124) *p* = 0.35	0.005 (–0.004 to 0.014) *p* = 0.32	

**Model 2**	**AUC (95% CI)**	**Brier (95% CI)**	**C-index**

**(C)**			
HFrEF	0.794 (0.709–0.878)	0.268 (0.187–0.349)	74.5
HFpEF	0.814 (0.717–0.911)	0.300 (0.208–0.391)	81.1
Difference	0.020 (–0.056 to 0.097) *p* = 0.60	0.032 (0.019–0.045) *p* < 0.001	

The list of variables for prediction of mortality using cumulative exposure to risk factors with associated sHR for CV and non-CV mortality are presented in Table 2 (Model 2). In this model, older age (1.05 per year [1.02–1.09]), history of RCA (4.8 [2.7–8.5]), history of MI (1.5 [0.9–2.5]), higher cumulative exposure to total cholesterol (1.3 per SD [1.1–1.7]), higher cumulative exposure to serum glucose (1.3 per SD [1.0–1.6]), longer QRS duration (1.3 per SD [1.0–1.6]), higher LVM (1.5 per SD [1.1–1.9]), higher NT-proBNP (1.1 per SD [0.9–1.4]), and statin use (0.7 [0.4–1.1]) were predictors of CV mortality after HF.

**FIGURE 3:**
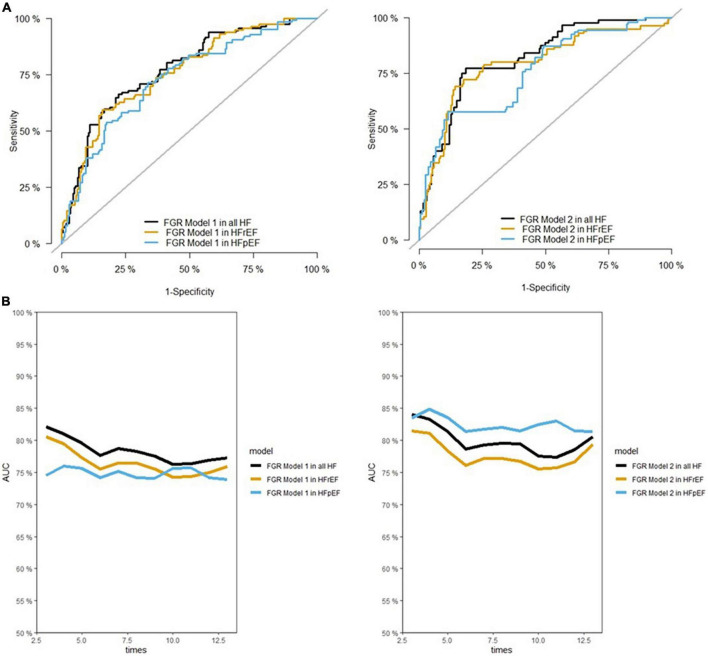
Area under the ROC (receiver operating characteristic) curve (AUC) for different models at maximum time of follow-up **(A)**, and during the follow-up **(B)**. FGR, fine-gray regression. As illustrated in panel **(B)**, the AUC of models were acceptable during the entire follow-up time.

The prediction performance (AUC: 0.82, *p* = 0.02) was higher and prediction accuracy slightly greater (Brier score 0.29, *p* = 0.26), although not significantly different when compared to the non-cumulative analysis. The predictive performance of this model was consistently high in both participants with HFrEF and HFpEF, with a higher prediction accuracy in patients with HFrEF (*p* < 0.001, Table 3B) than in those with HFpEF. Figure 3 presents the ROC curves and AUCs for the tested models over time in all participants with HF and in each HF subtype. As illustrated in Figure 3B, AUC of models were consistently high across the entire follow-up period.

## Discussion

In this study, we demonstrate that: (1) All-cause and CV mortality rates among participants with incident HFpEF are comparable to those with incident HFrEF, with prognostic prediction models having similar performances for both HF subtypes; (2) Age, pre-diagnosis history of RCA, MI, and diabetes, higher NT-proBNP levels, higher total cholesterol and lower HDL cholesterol concentrations, wider QRS, and LV hypertrophy were associated with greater CV mortality after HF; (3) Cumulative prior exposure to high total cholesterol and high serum glucose levels were associated with greater CV mortality; (4) In all participants with HF, as well as in those with HFpEF and those with HFrEF, there was a slight improvement—yet not significant—in predictive model performance when using cumulative prior exposure versus single timepoint measurements obtained before incident HF diagnosis.

Previous studies have shown higher rates of CV mortality in participants with HFrEF vs. HFpEF (25–27), whereas the rates of overall mortality did not differ between HF subgroups (25, 26). A study including over 2,500 participants with HF in the Framingham Heart Study (FHS) and Cardiovascular Health Study (CHS) showed that although the overall mortality rates in the two HF subtypes were not different, participants with HFrEF had a higher rate of CV death and participants with HFpEF had a higher rate of non-CV death, in both cohorts (26). We also observed similar all-cause mortality in patients with both HF subtypes; however, the CV mortality rate appeared to be greater in HFrEF participants although the difference was not statistically significant.

We used competing-risk analysis to specify the mortality attributable to CV versus non-CV etiologies. The predictive models in this study are applicable to all patients with incident HF. We observed robust predictive performances for both HF subgroups. On the other hand, the lack of CV mortality differences between the two subtypes could result from absence of effective therapeutic options in patients with HFpEF (28, 29). While detailed measures of cardiac remodeling were lacking in most of the previous studies (30), in our study, the absence of differences persisted after control for CMR-based myocardial anatomical parameters considered as best surrogates for cardiac remodeling (31).

The association of age and history of chronic diseases with poor HF prognosis has been previously documented (4, 7, 15). Older age is associated with declined contractility, prolongation of cardiac contraction, reduced compliance due to myocardial stiffness, and vascular stiffness (32). Also, a higher prevalence of concomitant organ dysfunction in older adults, as well as drug-disease interactions, partly account for worse outcomes in patients with HF (32). Our findings are consistent with the literature in that history of RCA, which was rare, prior MI, and presence of diabetes were prognostic of CV death. Similarly, previous studies have shown that history of cerebro- and cardiovascular diseases, along with other comorbidities, were significant predictors of mortality at 30-day and 1-year follow-up in patients with HF (33).

Prior reports have also shown that QRS interval prolongation is associated with an increased risk of mortality, particularly among African Americans (34). The risk of CV death, cardiac arrest, and hospitalization in patients with HFpEF was shown to increase linearly with QRS duration above 120 ms (35). Our study observed similar effect sizes for the association of QRS prolongation with HF mortality but of borderline statistical significance, likely due to the smaller sample size. These associations were attributed to conduction abnormalities, including left bundle branch block, frequently found in HF patients, especially in those with ischemic heart disease (36, 37).

LV hypertrophy is a strong predictor of incident HF and other CVD in MESA and other cohorts (18, 31). Our analysis demonstrated that LV hypertrophy is a strong predictor of CV mortality in patients with incident HF. This finding further supports the need for controlling risk factors such as hypertension to prevent or forestall LV hypertrophy and reduce mortality in patients with incident HF (38).

NT-proBNP is not only a risk variable for HF development (39) but also a predictor of adverse events in patients with HF, including mortality (40). At the subclinical level, higher NT-proBNP is associated with a greater decline in cardiac function over the years, independent of other risk factors (41). The prognostic value of this marker applies to both HFrEF and HFpEF (42, 43) and in this study, NT-proBNP appeared in both models as a top predictor of cardiovascular mortality.

The role of cumulative prior exposure to biomarkers in event prediction after the diagnosis of HF has not been investigated in detail. To our knowledge, this is the first study using cumulative prior exposure to risk factors in predictive models of cause-specific mortality in HF patients, with a mean follow-up of 4.7 years. MESA, with multiple measurements of metabolic biomarkers over time, provides a unique infrastructure for such analyses. The appearance of cumulative prior exposure to risk factors among the selected predictors, along with higher predictive performance in models including the cumulative prior exposure to risk factors emphasizes the importance of long-term control of dyslipidemia and hyperglycemia in HF prevention and reduction of post-HF mortality. Prior cumulative exposure to high serum glucose substituted for diabetes status our predictive models, emphasizing the value of long-term glucose maintenance. In practice, long-term exposure to such risk factors can be assessed using the Electronic Medical Records (EMR) enabling earlier and stricter risk factor control to reduce CV mortality in patients with HF.

Several limitations must be considered when interpreting the findings of this study. The limited overall number of incident HF events and further break-down into HF subgroups impacted our power to rule out small differences between subgroups. Therefore, the variable selection was performed on the overall sample, and models were applied on the two subgroups to assess the subtype-based prediction performance. The total number of MESA participants who developed HF was relatively small, perhaps secondary to the exclusion of participants with a history of heart disease at MESA at cohort inception, leading to an overall healthier cohort than the general population.

Moreover, in competing risk analysis, it is important to consider the effect sizes, event rates, and relativity of hazards, since the hazard of one endpoint can affect the other (44). This might manifest as opposite sHRs for the two competing risk endpoints. Therefore, from our analysis, it should not be concluded that higher cholesterol and NT-proBNP levels are protective for non-CV mortality.

Another limitation of this study is that we did not take into account the post-HF predictors of mortality, including therapeutic strategies used in these patients, which are major determinants of prognosis in patients with HF (45). In this regard, we assume that all participants were treated with the best therapeutic strategies after HF development. The findings of this study do not recommend routine measurement of CV imaging markers like LVM in healthy individuals. However, our results indicate that in patients with available prior CV imaging, morphological and functional markers can be used for predicting the risk of CV mortality at the time of HF diagnosis. Finally, another limitation of this study is that we did not know whether the participant had acute versus chronic HF at the time of death, and this status may determine different prognostic predictors (46).

## Conclusion

In summary, our study demonstrates similar all-cause and CV mortality rates in patients with incident HFrEF and incident HFpEF. High performance CV mortality prediction models were obtained for individuals who develop HF using data obtained at the latest time prior to HF diagnosis, as well as on cumulative prior exposure to risk factors before HF diagnosis. Predictors of CV mortality included age, pre-diagnosis history of RCA and MI, history of diabetes or cumulative prior exposure to high serum glucose levels, higher NT-proBNP levels, higher total cholesterol, and lower HDL cholesterol concentrations, wider QRS, and LV hypertrophy. Implementing cumulative exposure to risk factors, instead of latest measures, improves predictive accuracy for HF mortality, though not reaching statistical significance. Further studies in larger samples of patients with HF are needed to establish the predictive power of these variables for clinical implementation. This study aids physicians to tailor a more personalized treatment strategy earlier in the course of heart failure using their available medical records to estimate the risk and prevent mortality due to cardiovascular causes in long term.

## Data availability statement

The original contributions presented in this study are included in the article/Supplementary material, further inquiries can be directed to the corresponding author.

## Ethics statement

The studies involving human participants were reviewed and approved by the Johns Hopkins Medicine Institutional Review Board as part of the Multi-Ethnic Study of Atherosclerosis (MESA). The patients/participants provided their written informed consent to participate in this study.

## Author contributions

MS, MO, HB, JL, and DB: study design. RM, KL, SS, GB, WP, KW, AF, DB, and JL: data acquisition. MS, MO, XM, and CW: statistical analysis. MS, MO, XM, and HB: manuscript draft. HB, HC, MO, RM, KL, SS, GB, WP, KW, AF, DB, and JL: draft revision. JL, DB, and CW: supervision. All authors contributed to the article and approved the submitted version.
