# Supplementary material for: Pre-diagnostic predictors of mortality in patients with heart failure: The multi-ethnic study of atherosclerosis
Source: Front Cardiovasc Med. 2022 Dec 21;9:1024031. doi: 10.3389/fcvm.2022.1024031 (PMC9812565; doi:10.3389/fcvm.2022.1024031)
Supplement: Supplementary file 1 [file Data_Sheet_1.DOCX]

## Supplementary material:

### Methods:

Standardized questionnaires were used to collect information about age, sex, race/ethnic background and cigarette smoking. A medication inventory was used to collect information on prescription and nonprescription medications. Cigarette smoking was categorized as current, former or never. Body mass index (BMI) was calculated using body weight in Kg as nominator and squared height (in meter squared) as denominator. Weight was rounded to nearest 0.5 Kg and height was rounded to the nearest 0.001 m. Systolic and diastolic blood pressures were defined as the average of the last two measurements out of three measurements in resting and seated position. Blood pressure measurements were performed by Dinamap model Pro 100 automated oscillometric sphygmomanometer (Critikon, Tampa, FL). Fasting glucose was obtained by a thin-film adaptation of the glucose oxidase method (Johnson & Johnson Clinical Diagnostics, Inc, Rochester, NY). Diabetes mellitus was defined as a fasting glucose of ≥126 mg/dL or use of hypoglycemic medication. High-density Lipoprotein (HDL) and total cholesterol were analyzed using fasting blood samples and Low-density Lipoprotein (LDL) was calculated by the Friedewald formula. Glomerular Filtration Rate (GFR) was estimated by MDRD equation using serum creatinine, which was measured by colorimetry (Johnson & Johnson Clinical Diagnostics, Rochester, New York, USA) and calibrated with the standards of Cleveland clinic.

All ECGs were recorded with MAC 1200 ECG machines (Marquette Electronics, Milwaukee, WI, USA) and were analyzed at the EPICARE Center (Wake Forest University, Winston-Salem, NC, USA). The methodology for analysis has been described elsewhere ^1^. All cardiac MRI exams were performed using 1.5 T devices (Avanto and Espree, Siemens Medical Systems, Erlangen, Germany; and Signa HD, GE Healthcare, Milwaukee, Wis) and transferred to the MRI core lab (Johns Hopkins Hospital, Baltimore, MD, USA) for analysis. Image analysis protocols for LV volume and mass measurements, and LA function have been previously described ^2, 3^. Electron-beam CT and four –detector row CT devices were used to acquire non-contrast cardiac CTs and the coronary artery calcium was quantified using Agatston method in the CT core lab (University of California, Los Angeles, CA, USA) ^4^.

CVD was considered a composite of MI, RCA, definite or probable angina, stroke, stroke-related death, CHD-related death, atherosclerosis-related death and CVD-related death after MESA baseline. (In this study, we have only included non-death CVD events as history of CVD).

The angina definition in MESA study included probable and definite diagnosis only in participants with reported symptoms. In addition to symptoms, a physician diagnosis of angina and medical treatment for angina was defined as probable angina. Evidence of CABG surgery or other revascularization procedure; 70% or greater obstruction on coronary angiography; or evidence of ischemia by stress tests or by resting ECG, was required for definite angina.

The diagnosis of MI required either abnormal cardiac biomarkers (two times upper limits of normal) regardless of pain or ECG findings; evolving Q waves regardless of pain or biomarker findings; or a combination of chest pain, and ST-T evolution or new LBBB, and biomarker levels 1-2 times upper limits of normal. Stroke consisted of rapid onset of a documented focal neurologic deficit lasting 24 hours, or if less than 24 hours, there was a clinically relevant lesion on brain imaging. Patients with focal neurologic deficits secondary to brain trauma, tumor, infection, or other non-vascular cause were excluded. Cause of death was assigned for potential CVD deaths through committee review as part of MESA.

AFib was recorded using hospital discharge International Classification of Diseases, Ninth Revision (ICD9) diagnosis codes for AF or atrial flutter (427·31 or 427·32). MESA ascertained hospital discharge ICD-9 codes and Centers for Medicare and Medicaid Services (CMS) inpatient hospital claims. AF events during a hospital stay with coronary artery bypass surgery or valve replacement surgery were not counted as incident events.

### Figures:

Figure S1. Illustration of variable estimation and the area-under-curve using the B-spline based method in two sample variables (systolic blood pressure (SBP) and total cholesterol).


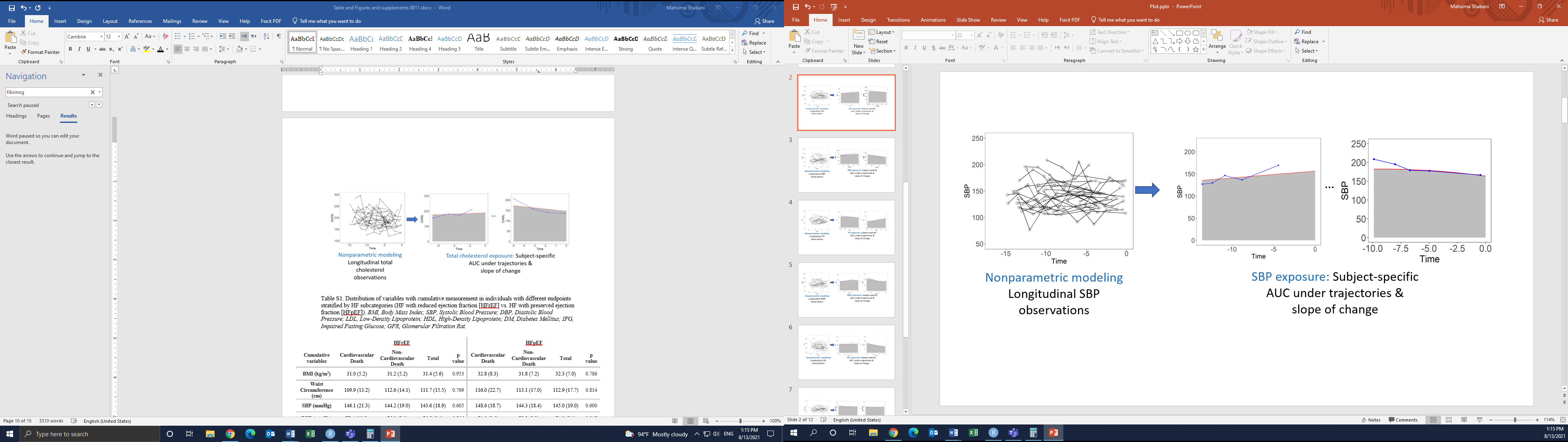


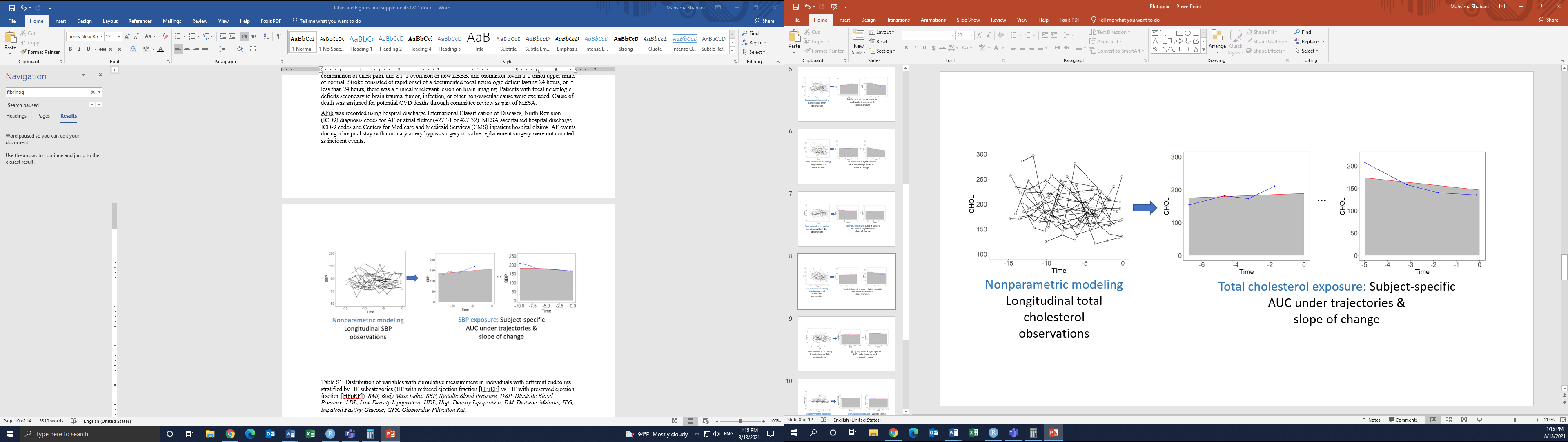


Figure S2. Study diagram illustrating the timing of incident HF with regards to MESA exams one to five. Year 0 is the baseline recruitment of participants in the MESA study (exam 1). The table below shows the number of HF-free population, with the entire 375 participants free of HF at the baseline.


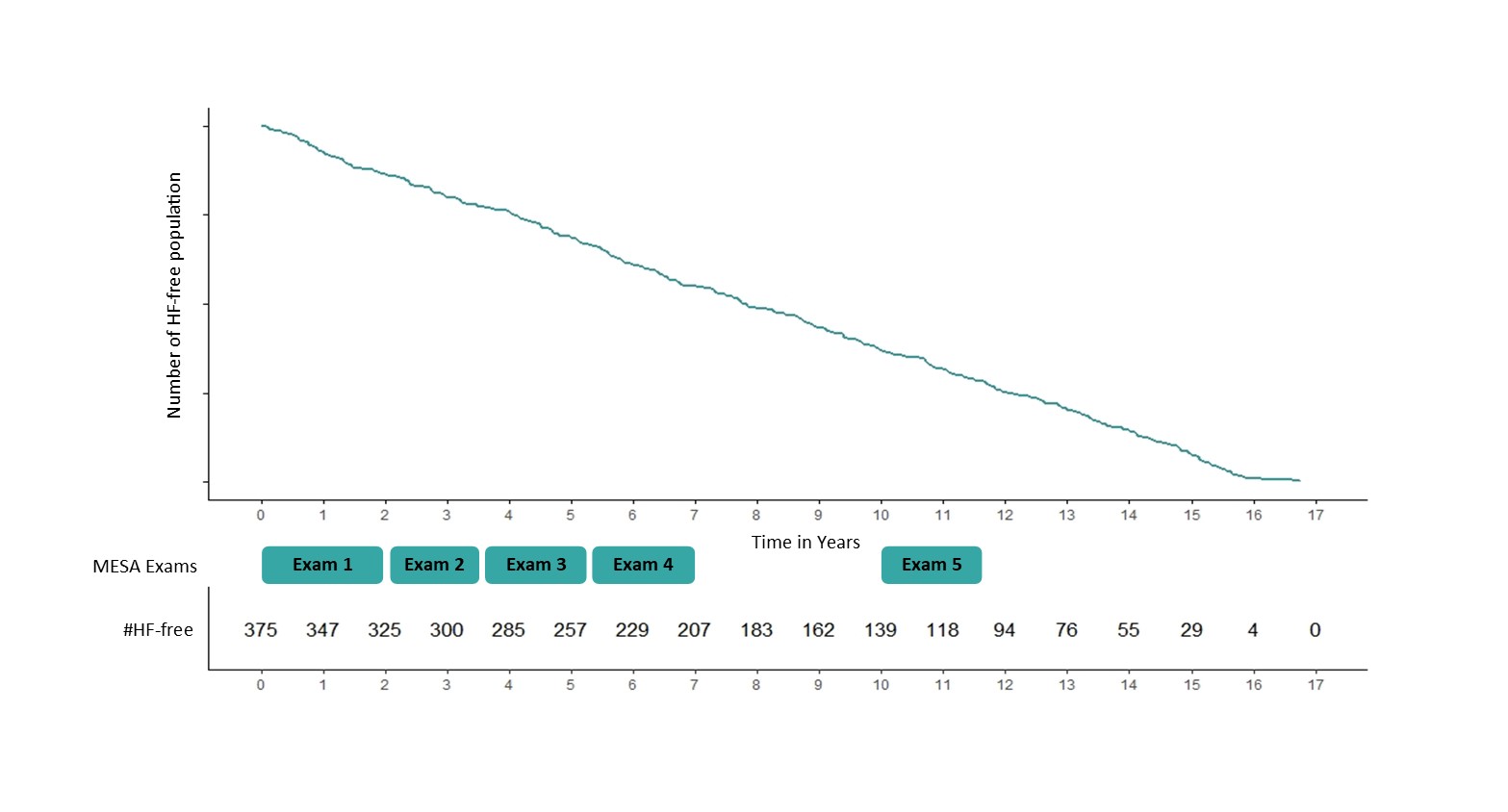


### Tables:

Table S1. Distribution of variables with cumulative measurement in individuals with different endpoints stratified by HF subcategories (HF with reduced ejection fraction [HFrEF] vs. HF with preserved ejection fraction [HFpEF]). *BMI, Body Mass Index; SBP, Systolic Blood Pressure; DBP, Diastolic Blood Pressure; LDL, Low-Density Lipoprotein; HDL, High-Density Lipoprotein; DM, Diabetes Mellitus; IFG, Impaired Fasting Glucose; GFR, Glomerular Filtration Rat.*

|  | HFrEF | | | | | HFpEF | | | | |
| --- | --- | --- | --- | --- | --- | --- | --- | --- | --- | --- |
| Cumulative variables | **Cardiovascular Death** | **Non-Cardiovascular Death** | **Total** | **p value** | **Cardiovascular Death** | | **Non-Cardiovascular Death** | **Total** | **p value** |  |
| BMI (kg/m^2^) | 31.0 (5.2) | 31.2 (5.2) | 31.4 (5.6) | 0.953 | 32.8 (8.3) | | 31.8 (7.2) | 32.3 (7.0) | 0.786 |  |
| Waist Circumference (cm) | 109.9 (13.2) | 112.6 (14.1) | 111.7 (15.5) | 0.769 | 116.0 (22.7) | | 113.1 (17.0) | 112.9 (17.7) | 0.814 |  |
| SBP (mmHg) | 146.1 (21.3) | 144.2 (19.0) | 143.6 (18.9) | 0.605 | 148.6 (18.7) | | 144.3 (18.4) | 145.0 (19.0) | 0.600 |  |
| DBP (mmHg) | 77.4 (10.0) | 76.3 (8.1) | 76.8 (9.1) | 0.766 | 74.0 (9.6) | | 73.2 (8.3) | 74.3 (8.4) | 0.347 |  |
| FBG (mg/dL) | 101.5 (18.2) | 106.0 (21.2) | 102.7 (19.7) | 0.335 | 115.3 (26.3) | | 102.8 (22.3) | 107.3 (24.9) | 0.491 |  |
| Triglyceride (mg/dL) | 119.8 (59.1) | 108.2 (38.2) | 115.0 (51.1) | 0.530 | 137.4 (79.1) | | 109.7 (45.6) | 123.6 (58.5) | 0.174 |  |
| LDL (mg/dL) | 123.0 (26.0) | 110.4 (23.1) | 116.6 (28.2) | 0.171 | 106.9 (26.1) | | 104.7 (23.6) | 109.9 (23.1) | 0.067 |  |
| HDL (mg/dL) | 46.7 (10.4) | 46.3 (9.4) | 48.7 (11.2) | **0.030** | 49.6 (12.0) | | 50.6 (14.4) | 50.2 (13.3) | 0.908 |  |
| Cholesterol (mg/dL) | 201.4 (30.3) | 186.4 (26.8) | 196.4 (35.6) | 0.089 | 190.6 (34.7) | | 184.0 (30.3) | 191.7 (31.9) | 0.139 |  |
| GFR (mL/min) | 79.5 (18.4) | 75.2 (24.1) | 79.0 (20.6) | 0.503 | 73.6 (19.3) | | 81.6 (23.9) | 79.4 (21.5) | 0.338 |  |

Table S2. Hazard ratio of cardiovascular death in univariate and age-adjusted competing risk models for each variable. BMI, Body Mass Index; CVD, Cardiovascular Diseases; Afib, Atrial Fibrillation; MI, Myocardial Infarction; RCA, Resuscitated Cardiac Arrest; COPD, Chronic Obstructive Pulmonary Disease; TIA, Transient Ischemic Attack; SBP, Systolic Blood Pressure; DBP, Diastolic Blood Pressure; FBG, Fasting Blood Glucose; LDL, Low-Density Lipoprotein; HDL, High-Density Lipoprotein; DM, Diabetes Mellitus; IFG, Impaired Fasting Glucose; GFR, Glomerular Filtration Rate, CAC, Coronary Artery Calcium, ABI, Ankle-Brachial Index; ACEI, Angiotensin-Converting-Enzyme Inhibitors; ARB, Angiotensin Receptor Blockers; LV, Left Ventricle; LA, Left Atrium; LVM, LV End-Diastolic Mass; EDV, End-Diastolic Volume; ESV, End-Systolic Volume; EF, Ejection Fraction.

|  |  | **Crude** | **Age-adjusted** |  |
| --- | --- | --- | --- | --- |
|  |  | **HR (95% CI) p value** | **HR (95% CI) p value** |  |
| **Age (years)** |  | **1.05 (1.02-1.08) p<0.001** | **-** |  |
| **Gender Male** |  | 1.21 (0.78-1.88) p=0.390 | 1.33 (0.86-2.05) p=0.200 |  |
| **Race** | Chinese American | 0.92 (0.35-2.37) p=0.860 | 0.95 (0.38-2.38) p:0.910 |  |
|  | African American | 1.42 (0.86-2.33) p=0.170 | **1.68 (1.02-2.77) p:0.040** |  |
|  | Hispanic | 1.28 (0.72-2.29) p=0.410 | 1.41 (0.79-2.50) p:0.240 |  |
| **BMI (kg/m^2^)** |  | 0.98 (0.94-1.01) p=0.210 | 0.99 (0.96-1.03) p=0.680 |  |
| **Waist circumference (cm)** |  | 0.88 (0.72-1.09) p=0.260 | 0.95 (0.76-1.18) p=0.620 |  |
| **Cigarette smoking** | Former | 1.04 (0.65-1.65) p=0.880 | 0.94 (0.59-1.50) p=0.800 |  |
|  | Current | 1.50 (0.75-3.01) p=0.250 | **2.18 (1.08-4.41) p=0.030** |  |
| **Education** | Higher than 12^th^ grade | 0.86 (0.57-1.32) p=0.505 | 0.91 (0.59-1.39) p=0.657 |  |
| **Log Physical activity (moderate and rigorous)** |  | 0.93 (0.83-1.04) p=0.183 | 1.02 (0.99-1.05) p=0.067 |  |
| **Medical History** | | | | |
| **History of Afib** |  | 0.65 (0.34-1.24) p=0.190 | 0.67 (0.35-1.29) p=0.230 |  |
| **History of stroke** |  | **3.23 (1.98-5.28) p<0.001** | **2.88 (1.71-4.84) p<0.001** |  |
| **History of MI** |  | **2.06 (1.35-3.16) p<0.001** | **1.90(1.23-2.95) p=0.004** |  |
| **History of RCA** |  | **4.51 (2.70-7.52) p<0.001** | **4.25 (2.49-7.26) p<0.001** |  |
| **History of COPD** |  | 0.86 (0.47-1.57) p=0.620 | 0.90 (0.49-1.64) p=0.730 |  |
| **History of TIA** |  | **3.94 (2.33-6.66) p<0.001** | **3.58 (2.06-6.21) p<0.001** |  |
| **Metabolic RFs** | | | | |
| **SBP (mmHg)** |  | 1.27 (1.00-1.62) p=0.050 | **1.28 (1.00-1.62) p=0.048** |  |
| **DBP (mmHg)** |  | 1.17 (0.89-1.54) p:0.270 | **1.34 (1.04-1.73) p=0.026** |  |
| **DM** | IFG | 1.50 (0.87-2.61) p=0.150 | 1.43 (0.83-2.48) p=0.200 |  |
|  | Controlled | 0.77 (0.20-2.95) p=0.700 | 0.74 (0.19-2.85) p=0.670 |  |
|  | Uncontrolled | 1.35 (0.82-2.21) p=0.240 | 1.50 (0.91-2.48) p=0.110 |  |
| **FBG (mg/dL)** |  | 0.96 (0.76-1.21) p=0.710 | 0.97 (0.77-1.23) p=0.820 |  |
| **LDL (mg/dL)** |  | 1.02 (0.82-1.28) p=0.850 | 1.10 (0.86-1.40) p=0.450 |  |
| **HDL (mg/dL)** |  | 0.96 (0.80-1.16) p=0.690 | 0.92 (0.76-1.11) p=0.390 |  |
| **Triglyceride (mg/dL)** |  | 1.11 (0.89-1.38) p=0.370 | 1.22 (0.97-1.52) p=0.087 |  |
| **Cholesterol (mg/dL)** |  | 1.05 (0.85-1.29) p=0.640 | 1.12 (0.90-1.39) p=0.310 |  |
| **Metabolic syndrome** |  | 1.16 (0.75-1.78) p=0.510 | 1.18 (0.77-1.81) p=0.460 |  |
| **GFR (mL/min)** |  | **0.80 (0.65-0.98) p=0.030** | 0.86 (0.69-1.06) p=0.160 |  |
| **CRP** |  | 0.99 (0.98-1.01) p=0.819 | 0.99 (0.98-1.01) p=0.673 |  |
| **Fibrinogen** |  | 1.00 (0.99-1.00) p=0.507 | 1.03 (0.99-1.05) p=0.060 |  |
| **Troponin (log)** |  | 1.10 (0.60-2.01) p=0.754 | 1.03 (1.00-1.05) p=0.574 |  |
| **NT-proBNP** |  | 0.99 (0.99-1.00) p=0.293 | 0.99 (0.99-1.00) p=0.282 |  |
| **CAC (log [agatston score])** |  | **1.13 (1.02-1.25) p=0.025** | 1.07 (0.96-1.20) p=0.210 |  |
| **ABI** |  | **0.15 (0.06-0.40) p<0.001** | **0.21 (0.07-0.61) p=0.006** |  |
| **Medication** | | | | |
| **Statin** |  | 0.75 (0.48-1.17) p=0.200 | 0.65 (0.42-1.02) p=0.062 |  |
| **ASA** |  | 0.93 (0.61-1.43) p=0.740 | 0.84 (0.55-1.30) p=0.440 |  |
| **ACEI** |  | 1.11 (0.70-1.75) p=0.660 | 1.19 (0.75-1.88) p=0.450 |  |
| **ACEI + Diuretic** |  | 0.73 (0.34-1.59) p=0.430 | 0.62 (0.29-1.34) p=0.230 |  |
| **ARB** |  | 0.84 (0.46-1.52) p=0.570 | 0.81 (0.45-1.44) p=0.470 |  |
| **ARB + Diuretic** |  | 0.94 (0.52-1.72) p=0.850 | 0.84 (0.46-1.52) p=0.560 |  |
| **NTG** |  | 0.45 (0.19-1.11) p=0.083 | **0.38 (0.16-0.90) p=0.027** |  |
| **Diuretics** |  | 0.85 (0.54-1.34) p=0.490 | 0.80 (0.51-1.26) p=0.340 |  |
| **Beta blocker** |  | 0.91 (0.56-1.47) p=0.700 | 0.89 (0.55-1.44) p=0.640 |  |
| **HTN med** |  | 1.27 (0.75-2.15) p=0.370 | 1.13 (0.67-1.92) p=0.640 |  |
| **Anti-coagulant** |  | 0.71 (0.36-1.41) p=0.330 | 0.62 (0.32-1.21) p=0.160 |  |
| **ECG** | | | | |
| **PR duration (ms)** |  | **1.12 (1.02-1.22) p=0.014** | 1.11 (1.00-1.23) p=0.050 |  |
| **QRS duration (ms)** |  | 1.17 (0.99-1.39) p=0.059 | 1.14 (0.95-1.36) p=0.150 |  |
| **QTc duration (ms)** |  | 1.07 (0.89-1.29) p=0.440 | 1.04 (0.87-1.25) p=0.660 |  |
| **CMR** | | | | |
| **LVM (g/m^2^)** |  | **1.37 (1.19-1.58) p<0.001** | **1.40 (1.22-1.60) p<0.001** |  |
| **LVEDV (mL/m^2^)** |  | **1.23 (1.06-1.43) p=0.007** | **1.27 (1.10-1.46) p<0.001** |  |
| **LVESV (mL/m^2^)** |  | **1.24 (1.07-1.44) p=0.004** | **1.29 (1.12-1.48) p<0.001** |  |
| **LVEF (%)** |  | **0.78 (0.65-0.94) p=0.008** | **0.75 (0.62-0.90) p=0.002** |  |
| **LA minimum volume** |  | 0.98 (0.96-1.01) p=0.255 | 0.99 (0.96-1.01) p=0.326 |  |
| **LA maximum volume** |  | 0.99 (0.97-1.01) p=0.419 | 0.99 (0.97-1.01) p=0.454 |  |
| **LA Active EF (%)** |  | 0.89 (0.73-1.08) p=0.240 | 0.90 (0.74-1.09) p=0.270 |  |
| **LA Passive EF (%)** |  | 0.87 (0.72-1.06) p=0.160 | 0.90 (0.74-1.10) p=0.310 |  |
| **LA total EF (%)** |  | 0.85 (0.69-1.03) p=0.100 | 0.87 (0.71-1.06) p=0.160 |  |

### References:

1. Ilkhanoff L, Soliman EZ, Ning H, Liu K, Lloyd-Jones DM. Factors associated with development of prolonged qrs duration over 20 years in healthy young adults: The coronary artery risk development in young adults study. *J Electrocardiol*. 2012;45:178-184

2. Natori S, Lai S, Finn JP, Gomes AS, Hundley WG, Jerosch-Herold M, Pearson G, Sinha S, Arai A, Lima JAC, Bluemke DA. Cardiovascular function in multi-ethnic study of atherosclerosis: Normal values by age, sex, and ethnicity. *American Journal of Roentgenology*. 2006;186:S357-S365

3. Habibi M, Samiei S, Ambale Venkatesh B, Opdahl A, Helle-Valle TM, Zareian M, Almeida AL, Choi EY, Wu C, Alonso A, Heckbert SR, Bluemke DA, Lima JA. Cardiac magnetic resonance-measured left atrial volume and function and incident atrial fibrillation: Results from mesa (multi-ethnic study of atherosclerosis). *Circ Cardiovasc Imaging*. 2016;9

4. Carr JJ, Nelson JC, Wong ND, McNitt-Gray M, Arad Y, David R. Jacobs J, Sidney S, Bild DE, Williams OD, Detrano RC. Calcified coronary artery plaque measurement with cardiac ct in population-based studies: Standardized protocol of multi-ethnic study of atherosclerosis (mesa) and coronary artery risk development in young adults (cardia) study. *Radiology*. 2005;234:35-43
